# Supplementary material for: Cell volume changes contribute to epithelial morphogenesis in zebrafish Kupffer’s vesicle
Source: eLife. 2018 Jan 29;7:e30963. doi: 10.7554/eLife.30963 (PMC5800858; doi:10.7554/eLife.30963)
Supplement: Figure 3—source data 1. — Here, we show the false negative rate β that results from testing against the alternative hypothesis that the true AP difference in a given case was the same as for the DMSO control at 8ss (Figure 3A). The statistical power in each case is 1-β. [file elife-30963-fig3-data1.docx]

| False negative rate β | AP cell volume differences | | AP LWR differences | |
| --- | --- | --- | --- | --- |
|  | 2 ss | 8 ss | 2 ss | 8 ss |
| DMSO control (Figure 3A) | 6% | -- | 0.5% | -- |
| Ouabain (Figure 3B) | 13% | 4% | 3% | 0.1% |
| CFTRinh (Figure 3C) | 9% | 2% | 0.9% | 0.08% |
| *cftr* MO (Figure 3D) | 10% | 12% | 1.4% | 3% |
