# Supplementary material for: Cell volume changes contribute to epithelial morphogenesis in zebrafish Kupffer’s vesicle
Source: eLife. 2018 Jan 29;7:e30963. doi: 10.7554/eLife.30963 (PMC5800858; doi:10.7554/eLife.30963)
Supplement: Figure 4—source data 1. — The cell volume is proportional to the product of cross-sectional area and height. As a consequence, if the AP difference in cell volume was fully due to the difference in the cross-sectional areas, then the percentage differences of both volumes and cross-sectional areas should be the same. Similarly, if the cell volume difference was fully due to the height difference, then the percentage differences of volumes and height should be the same. We find that at 2 ss, the AP differences are not high in all three quantities. However, at 8 ss the AP volume difference is very large and is mostly accounted for by the AP difference in cross-sectional areas, while the height difference stays comparably small. [file elife-30963-fig4-data1.docx]

| Relative anteroposterior (AP) differences of | 2 ss | 8 ss |
| --- | --- | --- |
| Cell volume | (-7 ± 22)% | (90 ± 40)% |
| Cell cross-sectional area | (11 ± 17)% | (80 ± 30)% |
| Cell height | (-1 ± 20)% | (18 ± 14)% |
