# Supplementary material for: Cell volume changes contribute to epithelial morphogenesis in zebrafish Kupffer’s vesicle
Source: eLife. 2018 Jan 29;7:e30963. doi: 10.7554/eLife.30963 (PMC5800858; doi:10.7554/eLife.30963)
Supplement: Figure 4—source data 2. — The listed values for lumen and KV cells are experimentally measured average cross-sectional areas from DMSO-treated control embryos. The 2 ss values for the external cells were set to the average of KV-ant and KV-post cells. The preferred area of the external cells at 8 ss is chosen such that the total preferred area stays constant between 2 ss and 8 ss. Note that in Figure 4A,B,E, the lumen area changes between 2 ss and 8 ss (same in Figure 4—figure supplements 2 and 3A,B,E), while in Figure 4C,D,F, the lumen area at 8 ss is set to the value measured at 2 ss (same in Figure 4—figure supplements 2 and 3C,D,F). In order to facilitate comparison with experimental data, we choose micro-meters (μm) as length units for our vertex model simulations. [file elife-30963-fig4-data2.docx]

| Cell type/lumen | Preferred area A_0_  at 2 ss (μm^2^) | Preferred area A_0_  at 8 ss (μm^2^) |
| --- | --- | --- |
| KV-ant cell | 199 | 238 |
| KV-post cell | 179 | 134 |
| External cell | 189 | 170 (lumen area changes) |
|  |  | 190 (no lumen area change) |
| Lumen | 1657 | 3602 (lumen area changes) |
|  |  | 1657 (no lumen area change) |
