# Supplementary material for: Cell volume changes contribute to epithelial morphogenesis in zebrafish Kupffer’s vesicle
Source: eLife. 2018 Jan 29;7:e30963. doi: 10.7554/eLife.30963 (PMC5800858; doi:10.7554/eLife.30963)
Supplement: Figure 6—source data 1. — Here, we show the false negative rate β that results from testing against the alternative hypothesis that the true AP difference in a given case was the same as for the MO control at 8 ss (Figure 6A). The statistical power in each case is 1-β. [file elife-30963-fig6-data1.docx]

| False negative rate β | Anteroposterior (AP) cell volume differences | Anteroposterior (AP) LWR differences |
| --- | --- | --- |
|  | 2 ss | 2 ss |
| Control MO (Figure 6A) | 0.005% | 0.00007% |
| *jupa* MO-1 (Figure 6B) | 0.2% | 0.02% |
| *lgl2* MO (Figure 6C) | 8% | 1% |
